# Supplementary material for: ACE Inhibitory Peptide from Skin Collagen Hydrolysate of Takifugu bimaculatus as Potential for Protecting HUVECs Injury
Source: Mar Drugs. 2021 Nov 24;19(12):655. doi: 10.3390/md19120655 (PMC8703921; doi:10.3390/md19120655)
Supplement: Supplementary file 1 [file marinedrugs-19-00655-s001.zip › marinedrugs-1458977-supplementary.pdf]

ACE Inhibitory Peptide from Skin Collagen Hydrolysate of *Takifugu bimaculatus* as Potential for Protecting HUVECs Injury

Shuilin Cai [1,2], Nan Pan [2], Min Xu [2,3], Yongchang Su [1,2], Kun Qiao [2], Bei Chen [2], Bingde Zheng [1*], Meitian Xiao [1*], and Zhiyu Liu [2*]

[1] College of Chemical Engineering, Huaqiao University, Xiamen 361021, China
[2] Key Laboratory of Cultivation and High-value Utilization of Marine Organisms in Fujian Province, Fisheries Research Institute of Fujian, Xiamen 361013, China
[3] College of Food and Biological Engineering, Jimei University, Xiamen, 361021, China
* Correspondence: bingd.zheng@hqu.edu.cn (B. Z.); mtxiao@hqu.edu.cn (M. X.); 13906008638@163.com (Z. L.)

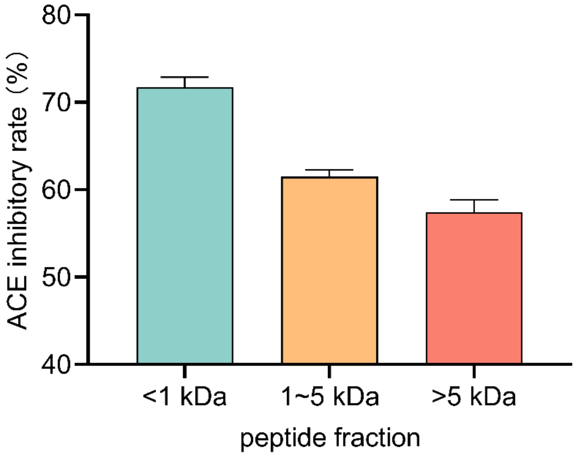

Figure S1. Effects of different peptide fractions on ACEI rate. TBSH-I (MW < 1 kDa), TBSH-II (1 kDa < MW < 5 kDa), and TBSH-III (MW > 5 kDa). The values were expressed as the means ± SD, n = 3. The mean ACEI rate was measured at 1 mg/mL.

Table S1. Peptide sequences identified by LC-MS/MS

NO	Peptide	ALC (%)	length	Mass	*m*/*z*	*z*	local confidence (%)
1	FAGF	96	4	440.2059	441.2133	1	96 92 99 99
2	FPSK	96	4	477.2587	478.2701	1	99 91 98 97
3	LAAF	98	4	420.2372	421.2439	1	98 97 100 99
4	LAPL	96	4	412.2686	413.2756	1	98 95 97 96
5	LCPT	95	4	432.2043	433.2112	1	98 93 95 97
6	LGPK	95	4	413.2638	414.2709	1	97 91 97 97
7	LPLP	96	4	438.2842	439.2913	1	93 97 99 98

Continued Table S1. Peptide sequences identified by LC-MS/MS

8	LPMP	97	4	456.2406	457.2477	1	98 96 99 98
9	LPPE	95	4	454.2427	455.2493	1	98 90 96 96
10	LVPF	97	4	474.2842	475.2921	1	97 96 99 96
11	LVPL	95	4	440.2999	441.3082	1	97 91 96 97
12	MVVP	95	4	444.2406	445.2476	1	94 95 98 95
13	PGLM	95	4	416.2094	417.2165	1	97 95 96 95
14	RAGP	95	4	399.223	400.2269	1	99 90 97 95
15	TLPP	95	4	426.2478	427.2552	1	95 92 98 99
16	VDDS	98	4	434.1649	435.1722	1	99 99 100 99
17	VGDF	95	4	436.1958	437.2031	1	93 88 99 99
18	VLAL	96	4	414.2842	415.2915	1	95 93 99 99
19	VLLL	97	4	456.3311	457.3386	1	94 98 100 99
20	VPPL	95	4	424.2686	425.2755	1	96 92 98 94
21	VVGF	99	4	420.2372	421.2437	1	100 99 100 99
22	VVGW	99	4	459.2482	460.2547	1	100 99 100 99
23	WGPE	95	4	487.2067	488.2142	1	98 92 95 96
24	AVGPL	98	5	455.2744	456.2819	1	95 97 100 100 100
25	DLDTK	95	5	590.2911	591.2991	1	88 93 99 100 99
26	DMFPK	98	5	636.2941	637.3012	1	97 96 100 100 99
27	EPGVP	95	5	497.2485	498.2566	1	99 98 97 95 88
28	FFGGL	98	5	539.2744	540.2819	1	99 98 99 99 97
29	GPGVP	98	5	425.2274	426.2337	1	99 98 99 99 99
30	GPGYP	98	5	489.2223	490.2296	1	96 98 99 99 98
31	KPGSP	96	5	484.2645	485.2719	1	100 98 97 95 91
32	LGTVP	95	5	485.2849	486.2933	1	97 94 96 96 92
33	LGVGP	96	5	441.2587	442.266	1	95 94 97 98 97
34	LHLFK	97	5	656.4009	657.4071	1	92 98 100 99 98
35	LLAPP	96	5	509.3213	510.3284	1	94 92 99 98 99
36	LLPPL	98	5	551.3682	552.376	1	99 99 99 96 97
37	PGGPR	98	5	482.2601	483.2682	1	98 98 99 99 99
38	VGGPF	95	5	475.243	476.2506	1	99 98 97 92 92

Continued Table S1. Peptide sequences identified by LC-MS/MS

NO	Peptide	ALC (%)	length	Mass	*m/z*	*z*	local confidence (%)
39	VGGPY	99	5	491.238	492.2452	1	99 100 100 100 100
40	VVGPF	95	5	517.29	518.2969	1	97 96 99 98 88
41	VVGPL	96	5	483.3057	484.313	1	100 99 99 94 92
42	VVVNP	95	5	526.3115	527.3185	1	99 97 98 93 92
43	EVDELR	96	6	759.3763	760.3831	1	95 93 99 99 96 95
44	FDLLRF	95	6	809.4435	405.7294	2	95 97 99 95 90 95
45	FNLRMQ	97	6	807.4061	404.7106	2	98 93 98 98 98 97
46	FPFLFR	98	6	825.4537	413.7334	2	97 97 99 99 99 99
47	GLPSVP	96	6	568.322	569.3293	1	92 97 98 99 98 98
48	KLPDGE	96	6	657.3333	658.3407	1	100 99 98 95 90 96
49	KSPVVP	97	6	625.3799	626.3879	1	99 95 95 98 99 99
50	KYPLER	97	6	804.4493	403.2318	2	100 99 98 92 98 99
51	LGSPGR	96	6	585.3234	586.3307	1	98 96 97 95 97 99
52	PGPGPM	97	6	554.2523	555.2615	1	96 100 100 99 98 92
53	STPDLE	95	6	660.2966	661.3029	1	95 95 93 95 94 97
54	VGPSVP	98	6	554.3064	555.3124	1	96 96 99 100 100 99
55	WLPLFK	96	6	802.4741	402.2439	2	97 97 97 97 95 96
56	DDLVEPR	96	7	842.4134	422.2131	2	92 90 98 98 99 98 98
57	ELPVLLK	95	7	810.5214	406.268	2	85 84 99 100 100 100 100
58	ETAPGMP	96	7	701.3054	702.3119	1	99 98 98 97 95 91 96
59	FEGPGSP	97	7	689.302	690.3085	1	97 97 95 99 99 99 98
60	FHLPHGL	95	7	819.4391	410.7264	2	99 94 99 98 93 94 95
61	FPPDGLR	96	7	800.418	401.2152	2	94 94 94 97 98 99 98
62	KFDPVLR	98	7	873.5072	437.761	2	97 97 100 98 98 99 99
63	LALPWLK	95	7	839.5269	420.7708	2	89 89 99 97 97 99 98
64	LAPPERK	95	7	809.4759	405.7452	2	100 100 100 99 93 82 92
65	LDKVRFL	95	7	889.5385	445.7768	2	90 96 98 96 94 95 96
66	WKPPTDD	95	7	857.3919	429.7029	2	77 91 100 99 98 100 100
67	AGGYTRLL	98	8	849.4708	425.7425	2	99 99 99 98 98 98 99 100
68	EAAPLNPK	95	8	838.4548	420.2346	2	94 94 100 98 95 89 94 97

Continued Table S1. Peptide sequences identified by LC-MS/MS

NO	Peptide	ALC (%)	length	Mass	*m/z*	*z*	local confidence (%)
69	EGAPLNPK	95	8	824.4391	413.2268	2	82 85 100 100 99 97 98 99
70	GPGFPGER	95	8	815.3926	408.7035	2	95 97 96 95 96 87 95 98
71	LLAPPELR	96	8	907.5491	454.7819	2	92 95 100 99 96 97 97 96
72	LLAPPEVK	97	8	865.5273	433.7694	2	99 99 100 100 90 92 99 99
73	LLAPPEVR	98	8	893.5334	447.7736	2	98 98 99 99 97 98 97 98
74	QPGPPNPR	96	8	861.4457	431.7296	2	93 98 99 98 93 94 98 99
75	TLPTTSPK	95	8	843.4702	422.7421	2	90 84 99 99 99 98 98 99
76	LLPGNLLVR	98	9	993.6335	497.824	2	99 99 100 99 97 99 99 98 98
77	TLLPGLGKL	95	9	910.5851	456.2998	2	96 98 100 96 86 92 96 98 98
78	VGGPSPAGP	98	9	737.3708	738.3781	1	99 98 99 99 99 97 98 99 100
79	WFRDGQELR	96	9	1205.594	402.8715	3	100 99 99 100 98 90 97 94 94
80	YSPGASGPK	97	9	862.4185	432.2167	2	93 92 99 98 97 99 99 99 100
81	FERPDLLERP	96	10	1270.667	424.5629	3	99 99 97 96 99 100 98 95 92 95
82	PGSGPSPGAP	97	10	822.3871	823.3948	1	92 92 99 100 100 100 100 98 98 98

Continued Table S2. Sequences and score of potential bioactive peptides

Sequences	Docking Score (kcal/mol)	Sequence	Docking Score (kcal/mol)	Sequence	Docking Score (kcal/mol)
WFRDGQELR	-19.69	EGAPLNPK	-14.71	LGVGP	-11.46
LLAPPEVR	-18.47	TLPTTSPK	-14.44	VVGPF	-11.46
FERPDLLERP	-18.28	ETAPGMP	-14.26	GPGYP	-11.32
LLAPPELR	-17.27	LGSPGR	-14.08	VGGPY	-11.30
LLAPPEVK	-16.85	FPFLFR	-13.99	LVPF	-10.89
WKPPTDD	-16.71	LPPE	-13.80	LAPL	-10.84
ELPVLLK	-16.47	FHLPHGL	-13.74	VVGW	-10.81
TLLPGLGKL	-16.42	DLDTK	-13.61	MVVP	-10.80
LALPWLK	-16.37	WLPLFK	-13.56	VLAL	-10.76
EVDELR	-16.29	FEGPGSP	-13.43	FAGF	-10.48
FDLLRF	-16.27	VGGPSPAGP	-13.39	VVGPL	-10.46

Table S2. Sequences and score of potential bioactive peptides

FPPDGLR	-16.16	WGPE	-13.31	VVVNP	-10.43
STPDLE	-15.96	GLPSVP	-12.99	VLLL	-10.39
FNLRMQ	-15.80	KSPVVP	-12.97	FPSK	-10.32
KFDPVLR	-15.73	EPGVP	-12.92	LVPL	-10.20
LAPPERK	-15.70	LHLFK	-12.52	VPPL	-10.17
LLPGNLLVR	-15.67	VDDS	-12.47	RAGP	-10.15
PGSGPSPGAP	-15.67	PGPGPM	-12.12	LPLP	-10.12
KLPDGE	-15.66	LLPPL	-12.10	VVGF	-9.79
QPGPPNPR	-15.60	VGDF	-12.09	LAAF	-9.49
GPGFPGER	-15.59	VGPSVP	-11.91	LGPK	-9.49
YSPGASGPK	-15.48	AVGPL	-11.90	GPGVP	-9.44
EAAPLNPK	-15.40	FFGGL	-11.75	TLPP	-9.44
DDLVEPR	-15.27	LGTVP	-11.71	LCPT	-9.44
DMFPK	-15.18	PGGPR	-11.67	LPMP	-9.36
LDKVRFL	-15.14	LLAPP	-11.65	PGLM	-9.34
KYPLER	-14.83	VGGPF	-11.64		
AGGYTRLL	-14.82	KPGSP	-11.47		

Table S3. The sequences of chemically synthesized peptide with ACEI activity.

Sequence	ACEI rate（%）	Sequence	ACEI rate（%）	Sequence	ACEI rate（%）
WFRDGQELR	59.76±1.63	FPPDGLR	47.18±1.26	GPGFPGER	26.59±0.77
LLAPPEVR	35.36±0.97	STPDLE	21.29±0.78	YSPGASGPK	18.65±0.61
FERPDLLERP	28.21±1.12	FNLRMQ	80.35±1.81	EAAPLNPK	16.34±0.68
LLAPPELR	40.06±0.45	KFDPVLR	28.03±0.71	DDLVEPR	27.25±1.01
LLAPPEVK	44.39±0.54	LAPPERK	37.32±1.05	DMFPK	36.93±0.97
WKPPTDD	41.27±1.08	PGSGPSPGAP	34.86±1.05	LDKVRFL	31.86±0.94
TLLPGLGKL	36.59±0.83	KLPDGE	31.14±0.95		
EVDELR	29.73±0.58	QPGPPNPR	25.36±0.71		

Note: It is necessary to evaluate the feasibility of all peptide sequences before synthesis. Therefore, we assessed the feasibility of the synthesis process of these potential peptides with the help of an analyzing tool (https://www.genscript.com/tools/peptide-analyzing-tool). Finally, a total of 22 peptides were chemically synthesized and tested for ACEI activity. The values were expressed as the means ± SD, n = 3. The mean ACEI rate was measured at 1 mg/mL.

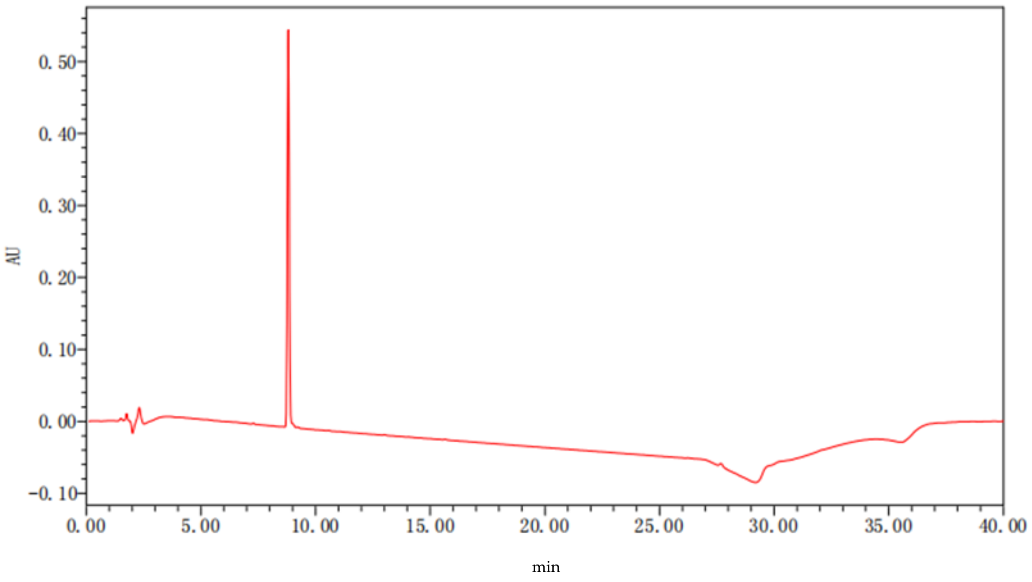

Figure S2. HPLC of synthetic peptide FNLRMQ

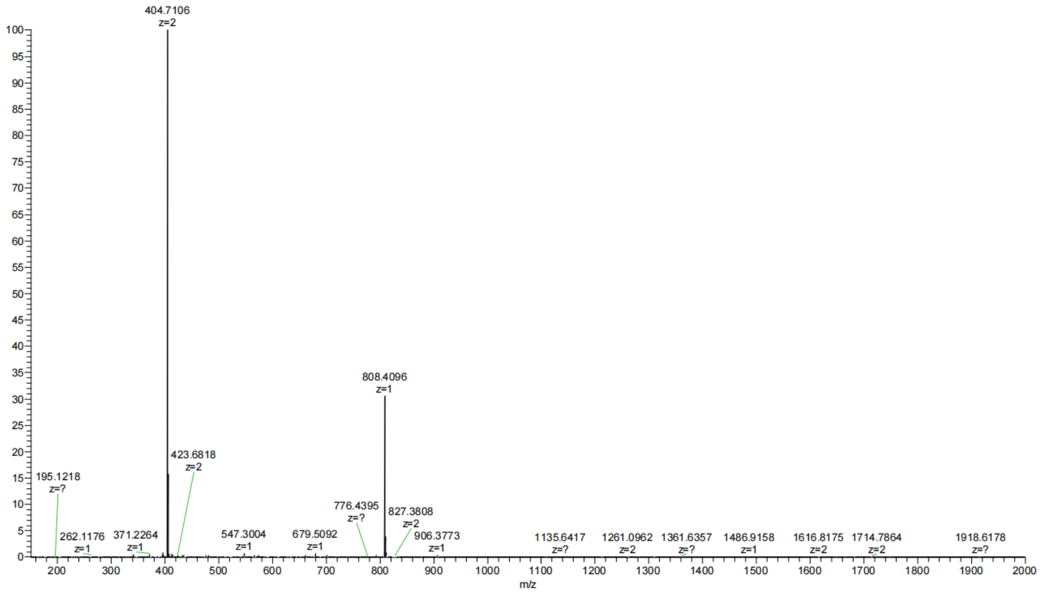

Figure S3. MS of synthetic peptide FNLRMQ

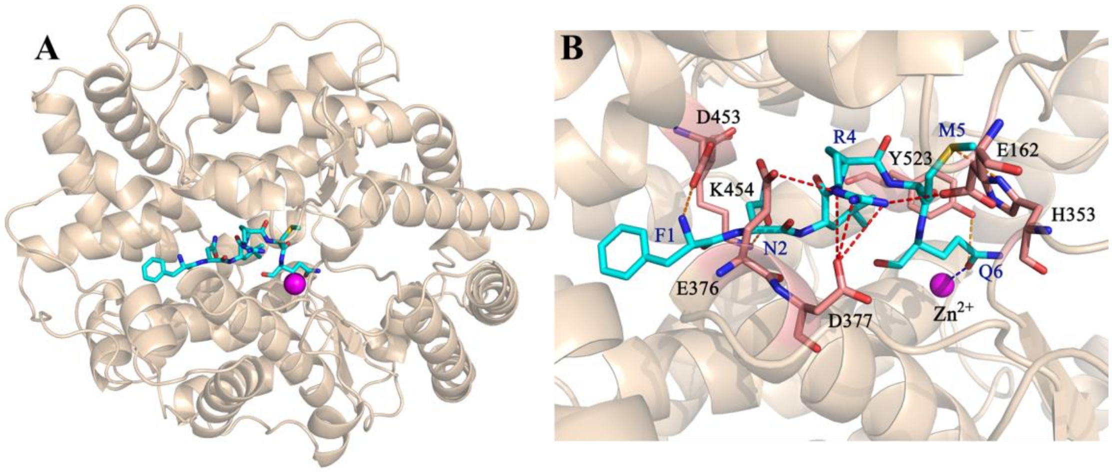

Figure S4. The binding mode of FNLRMQ with ACE. (A) The binding mode of FNLRMQ with ACE. (B) The detail binding mode of FNLRMQ with ACE. FNLRMQ is depicted as cyan stick, Zn^{2+} is depicted as a sphere (magenta). The backbone of ACE is depicted as wheat cartoon with transparency, the surrounding residues in the binding pockets are colored in pink. H bonds, salt bridges, and interactions with Zn^{2+} are shown as orange, red, and blue dashes lines

Table S4. The contact list between FNLRMQ and ACE.

ChainA	Residue	ChainB	Residue	Interaction type
ACE	His353	FNLRMQ	M5	Hydrogen bond interaction
ACE	Asp453	FNLRMQ	F1	Hydrogen bond interaction
ACE	Lys454	FNLRMQ	N2	Hydrogen bond interaction
ACE	Tyr523	FNLRMQ	Q6	Hydrogen bond interaction
ACE	Zn^{2+}	FNLRMQ	Q6	Ion contact
ACE	Glu162	FNLRMQ	R4	Salt bridge
ACE	Glu376	FNLRMQ	R4	Salt bridge
ACE	Asp377	FNLRMQ	R4	Salt bridge

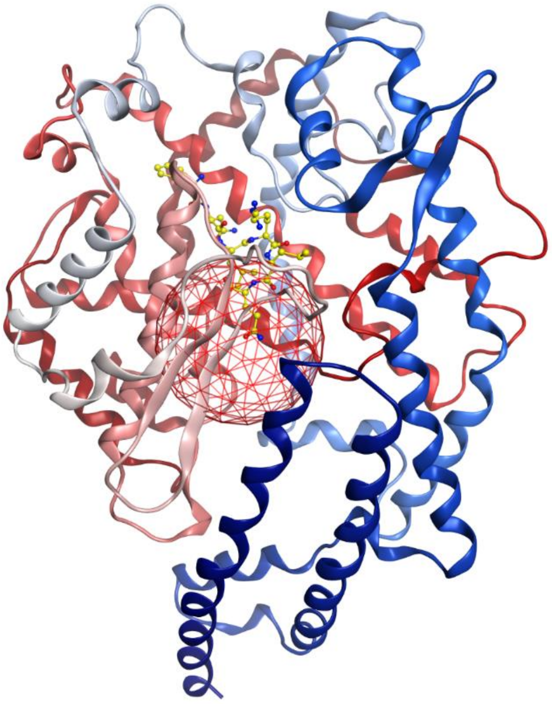

Figure S5. The binding pocket in protein ACE.

Note: Angiotensin I-converting enzyme (ACE, EC3.4.15.1, dipeptidyl carboxypeptidase) is a Zn^{2+}-dependent membrane-bounded protein that consisted of four characteristic sectors, namely, the signal peptide, functional domain, transmembrane structure and intracellular region [1]. Among them, the functional domain is subdivided into two different structural domains (N-domain and C-domain), and the two homologous structural domains are connected by 15 amino acid residues. Both homologous structural domains contain the Zn^{2+}-binding module HEXXH (H for His, histidine; E for Glu, glutamic acid; X for any amino acid residue) [1]. The catalytic region of the ACE structural domain contains three active pockets: S1 (Ala354, Glu384, and Tyr523), S2 (Gln281, His353, Lys511, and Tyr520) and S1′ (Glu 162) [2].

References

1. Natesh, R.; Schwager, S. L.; Sturrock, E. D.; Acharya, K. R., Crystal structure of the human angiotensin-converting enzyme-lisinopril complex. *Nature* 2003, 421, 551-4.
2. Spyroulias, G. A.; Galanis, A. S.; Pairas, G.; Manessi-Zoupa, E.; Cordopatis, P., Structural features of angiotensin-I converting enzyme catalytic sites: conformational studies in solution, homology models and comparison with other zinc metallopeptidases. *Curr. Top. Med. Chem.* 2004, 4, 403-29.
